# The Coherent X-ray Imaging instrument at the Linac Coherent Light Source

**DOI:** 10.1107/S160057751500449X

**Published:** 2015-04-15

**Authors:** Mengning Liang, Garth J. Williams, Marc Messerschmidt, M. Marvin Seibert, Paul A. Montanez, Matt Hayes, Despina Milathianaki, Andrew Aquila, Mark S. Hunter, Jason E. Koglin, Donald W. Schafer, Serge Guillet, Armin Busse, Robert Bergan, William Olson, Kay Fox, Nathaniel Stewart, Robin Curtis, Alireza Alan Miahnahri, Sébastien Boutet

**Affiliations:** a Linac Coherent Light Source, SLAC National Accelerator Laboratory, 2575 Sand Hill Road, Menlo Park, CA 94025, USA

**Keywords:** FEL, coherent diffracted imaging, serial femtosecond crystallography, protein crystallography, single molecule imaging

## Abstract

The Coherent X-ray Imaging (CXI) instrument specializes in hard X-ray, in-vacuum, high power density experiments in all areas of science. Two main sample chambers, one containing a 100 nm focus and one a 1 µm focus, are available, each with multiple diagnostics, sample injection, pump–probe and detector capabilities. The flexibility of CXI has enabled it to host a diverse range of experiments, from biological to extreme matter.

## Abstract 2

Description of the Coherent X-ray Imaging (CXI) instrument at the Linac Coherent Light Source. Recent scientific highlights illustrate the femtosecond crystallography, high power density and extreme matter capabilities of the CXI instrument.

## 1. Introduction   

The Coherent X-ray Imaging (CXI) instrument was constructed to pursue the goal of molecular imaging using coherent diffractive imaging (CDI) in the diffraction before destruction regime (Neutze *et al.*, 2000 ▶). The main requirements for this technique are: high flux to provide enough scattered photons from a small object to obtain sufficient signal-to-noise, coherence of the incident field and short pulse length, on the order of tens of femtoseconds, to outrun the radiation damage which would cause displacement from non-irradiated positions. CXI was designed specifically for high fluence, employing a pair of 1 µm Kirkpatrick–Baez (KB) mirrors and another pair to produce a 100 nm focus, intended to capture the full incident beam to maximize the power density on samples of varying sizes. Maximizing the number of photons incident on small targets is critical to study low-*Z* materials such as biomolecules. Additionally, CXI has flexible sample and detector geometries to support a wide variety of experiments, utilizing both facility-provided resources and those from user groups (Boutet & Williams, 2010 ▶).

With the goal of molecular imaging in mind, CXI has hosted experiments in imaging small compact objects, biological and fabricated, as well as X-ray protein crystallography of small crystals and time-resolved studies. These approaches address the many challenges of molecular imaging, from algorithm development, three-dimensional merging and sample delivery by utilizing the vast expertise of synchrotron X-ray protein crystallography and coherent diffractive imaging techniques.

## 2. Instrument overview   

**Figure 1:**
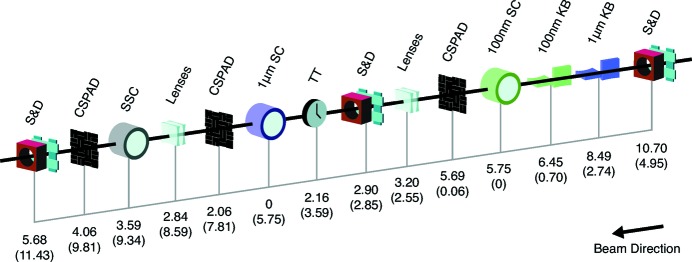
Overview of the CXI instrument layout. Distances are indicated in meters from the 1 µm sample chamber (SC) and in parentheses for the 100 nm sample chamber. Each chamber is colored to match its corresponding KB pair. One set of Be lenses can be used to further control the focus in the 1 µm sample chamber and another set to refocus the unscattered beam to the serial sample chamber at 3.59. There are slits and diagnostic (S&D) along the beamline and a timing tool (TT) for fine timing between optical laser and X-ray beams for pump–probe experiments. The sample is located approximately 440 m downstream of the undulators.

CXI is primarily an in-vacuum forward-scattering-geometry instrument, but it is sufficiently flexible to host a variety of hard X-ray experiments that benefit from short pulses and are best operated in vacuum. The unique capabilities of CXI have allowed for many types of experiments, including matter in extreme conditions, investigations of radiation damage, X-ray–matter interaction studies and structural biology. A schematic of major instrument components is shown in Fig. 1 ▶ and a list of instruments parameters is given in Table 1 ▶.

The X-ray beam which emerges from the undulators has many components in addition to the FEL beam, such as bremsstrahlung, gamma rays, broad spectrum undulator radiation including high-energy spontaneous emission. A set of mirrors in the front-end enclosure is used to filter undesired light, both spectrally and spatially (Soufli *et al.*, 2011 ▶). These mirrors reflect all photon energies below ∼25 keV with a gradual decrease in efficiency above that. The lowest usable energy of the mirrors is primarily determined by their finite length, with the lower photon energies having a much larger divergence causing the beam to greatly overfill the mirrors, making the use of these photon energies impractical and inefficient (Barty *et al.*, 2009 ▶). In practice, the low-energy cutoff is ∼4–5 keV. The beam that is delivered through over 300 m of vacuum pipe and a 220 m-long X-ray tunnel into CXI arrives with a FWHM of ∼750 µm at ∼8 keV with a strong dependence on the photon energy. CXI has four sets of slits which are used to define the mirror aperture in addition to minimizing scattering from optical components. These slits are made of Si_3_N_4_ owing to its ability to withstand the full LCLS beam in the typical range of operation of CXI, between 5 and 11 keV. Multiple diagnostics are available along the instrument, including profile–intensity monitors and intensity–position monitors (Feng *et al.*, 2011 ▶). Two sets of silicon attenuator stacks are available to tune the incident flux to the desired level.

CXI has one Be compound refractive lens (CRL) stack and two sets of KB mirror pairs to focus the beam to 10 µm, 1 µm and 100 nm, respectively. Be CRLs are in-line optics, with interchangeable sets providing the flexibility to focus at different energies and to different focal lengths. Three independent sets can be mounted at any given time, allowing three different photons energies that can be focused for any given experiment. The 1 µm KB mirrors, fabricated by JTEC with a positioning system from Bruker ASC, are SiC coated. They have a 90% reflectivity (Soufli *et al.*, 2011 ▶) and a 50–80% efficiency depending on the energy from 5 keV to 11 keV, with increased efficiency for higher photon energy owing to the smaller beam size at the mirrors. The maximum incidence angle at the downstream end of the mirrors is 3.4 mrad, leading to an ∼11 keV reflectivity cutoff, and the focal length for each mirror is 8.7 m and 8.5 m with an acceptance aperture of approximately 1.2 mm for the 1 µm KBs (Siewert *et al.*, 2012 ▶). The 100 nm KB mirrors are similar to the 1 µm mirrors, with a ten times higher curvature, focal lengths of 0.9 m and 0.5 m for each mirror and an acceptance aperture of 1.0 mm. The actual focused beam profiles are not Gaussian and have broad intensity tails which limit the maximum and average power density on the sample. A detailed analysis of the focused beam properties is ongoing.

CXI has two main experimental chambers, one centered at the 10 µm and 1 µm focus position, used for particles of the order of 1 µm in size or samples which would benefit from a larger area of illumination rather than higher peak intensity. The 1 µm chamber is compatible with two Cornell–SLAC pixel array detector (CSPAD) (Hart *et al.*, 2012 ▶) detector chambers which can be used in tandem to obtain high- and low-resolution data simultaneously. Experiments on smaller samples requiring high peak fluence can use the 100 nm sample chamber which is compatible with only one full size CSPAD but can also make use of a smaller CSPAD-140k for low-angle measurements. Both chambers contain an in-line visible-light optic to image the interaction region from the incident X-ray beam direction and breadboards which can be used to install additional apertures, stages, optics or diagnostics such as diodes or spectrometers. Standard sample delivery systems and a time-of-flight ion spectrometer are designed to be installed at the top of both chambers. Both chambers are also designed for laser in-coupling to facilitate pump–probe experiments. Small versions of the CSPAD, consisting of only 1/16 of the full CSPAD (Herrmann *et al.*, 2013 ▶) are available to be used for a variety of experiments and may be mounted in multiple locations along the instrument (Szlachetko *et al.*, 2014 ▶) and inside the sample chambers (Kern *et al.*, 2013 ▶).

Sample delivery is critical for FEL experiments owing to the type of samples and the high repetition rate of the machine. For biological systems, samples should ideally remain in an aqueous environment but with as little surrounding liquid as possible to reduce background scattering. To utilize the 120 Hz repetition rate of LCLS, the sample must be replenished at a corresponding rate. The two main systems, which are used at CXI, are liquid jets and aerosol sources. Among the liquid jets, the gas dynamic virtual nozzle (GDVN) (DePonte *et al.*, 2008 ▶) is the most commonly used. Other slower flowing jets such as an electrospinning source (Sierra *et al.*, 2012 ▶), a lipidic cubic phase jet (Weierstall *et al.*, 2014 ▶) as well as other high-viscosity carrier media (Botha *et al.*, 2015 ▶) are all used to form flowing columns of sample at various speeds. Aerodynamic lens sources focus an aerosol to create a high particle density at the interaction region (Bogan *et al.*, 2008 ▶). For systems which are not amenable to suspension and injection with either method, sample chips that have thin SiN windows with an aqueous layer sandwiched between them or other protection from drying in vacuum can be prepared (Nam *et al.*, 2013 ▶). Similarly, samples which do not require an aqueous environment can be mounted with a variety of sample stages in multiple configurations (Hunter *et al.*, 2014 ▶).

Core laser systems at the LCLS consist of an ultrashort pulse and a Ti:sapphire oscillator synchronized to the FEL, seeding a commercially available chirped pulse amplifier producing 4 mJ at 40 fs. A more in-depth description of the optical laser capabilities at LCLS is given by Minitti *et al.* (2015 ▶). While some other hutches at LCLS have home-built four-pass amplifiers that can boost the pulse energy to over 30 mJ, the CXI laser system currently does not and has a more limited pulse energy range, which also limits the available spectral range for femtosecond laser pulses. An optical parametric amplifier can be used to generate other wavelengths outside of the harmonics of the 800 nm source. Nd:YLF nanosecond laser systems are also available (Kern *et al.*, 2013 ▶; Kupitz *et al.*, 2014 ▶). The arrival time of the femtosecond lasers compared with the X-rays can be directly measured at CXI with high accuracy using a recently installed time-tool developed at LCLS and other FEL facilities (Harmand *et al.*, 2013 ▶).

In order to maximize use of the beam, a serial sample chamber (SSC) will be installed downstream of the first CSPAD for the 1 µm chamber to refocus the unscattered beam from the 1 µm chamber with Be lenses. The SSC will be used particularly for serial femtosecond crystallography and could as much as double the beam time at CXI when the 1 µm sample chamber is used without the need for a second detector to image the low-*q* regime.

## 3. Research highlights   

Pursuing the goal of molecular imaging, CXI has been used by a large user community to develop the technique of serial femtosecond nanocrystallography (SFX), which extends traditional X-ray protein crystallography to room temperature and dynamic studies as well as potentially smaller crystals. The technique merges thousands to tens of thousands of two-dimensional diffraction patterns to obtain a three-dimensional data set which can then be analyzed using standard protein crystal phasing techniques. The first experiments were performed on well known model systems (Chapman *et al.*, 2011 ▶; Boutet *et al.*, 2012 ▶) and, since the establishment of SFX, it has been widely applied to systems or dynamics that were difficult or impossible to study at room temperature using more conventional macromolecular crystallography approaches (Kern *et al.*, 2013 ▶; Kupitz *et al.*, 2014 ▶; Liu *et al.*, 2013 ▶; Demirci *et al.*, 2013 ▶).

**Figure 2:**
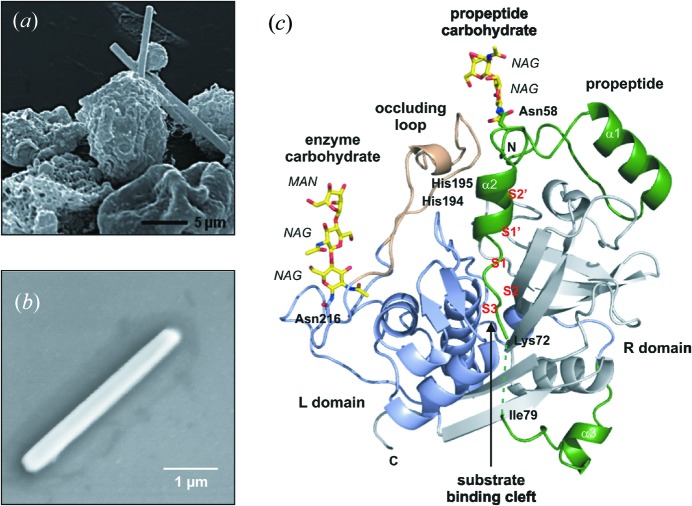
*In vivo* grown crystals and three-dimensional structure of the *T. brucei* cathepsin B–propeptide complex. (*a*) Scanning electron micrograph of a group of Sf9 insect cells infected with TbCatB virus 80 h after infection showing crystals of overexpressed TbCatB. (*b*) Scanning electron micrograph of a single TbCatB crystal after isolation. (*c*) Cartoon plot of the TbCatB–propeptide complex exhibiting the typical papain-like fold of cathepsin B-like proteases. Gray, R domain; blue, L domain; beige, occluding loop. The native propeptide (green) blocks the active site. Two N-linked carbohydrate structures (yellow) consist of *N*-acetylglucos­amine (NAG) and mannose (MAN) residues (yellow, carbon atoms; blue, nitrogen atoms; red, oxygen atoms). [From Redecke *et al.* (2013 ▶), *Science*, **339**, 227–230. Reprinted with permission from AAAS.]

*Trypanosoma brucei* cysteine protease Cathepsin B (TbCatB) is a protein that is a potential drug target to treat sleeping sickness. The glycosolated protein form is not amenable to the growth of large crystals needed for traditional crystallography, but forms *in vivo* with the expression of the protein in insect Sf9 cells infected with a bacculovirus (Koopmann *et al.*, 2012 ▶). The *in vivo* crystal size is limited to the physical dimension of the cell which makes it uniquely suited to SFX. The SFX experiments on TbCatB performed at CXI were on 0.9 µm × 0.9 µm × 11 µm crystals in a GDVN (Redecke *et al.*, 2013 ▶). Almost 300000 data frames from individual crystals were obtained with 61% successfully indexed to obtain the three-dimensional diffraction data set. The resulting molecular structure reveals the specific binding of the propeptide, which is the native inhibitor of TbCatB, together with a stabilizing impact of two carbohydrate structures unseen in the deglycosylated mature protein. An electron microscope image shows infected Sf9 cells (Fig. 2 ▶ *a*), a TbCatB crystal (Fig. 2 ▶ *b*) and the refined protein structure (Fig. 2 ▶ *c*). The structure of this natural specific inhibitor may provide drug designers with critical information as to how to target this protein in the treatment of the disease. This new structural information shows the potential for SFX at FELs to elucidate challenging systems at room temperature.

**Figure 3:**
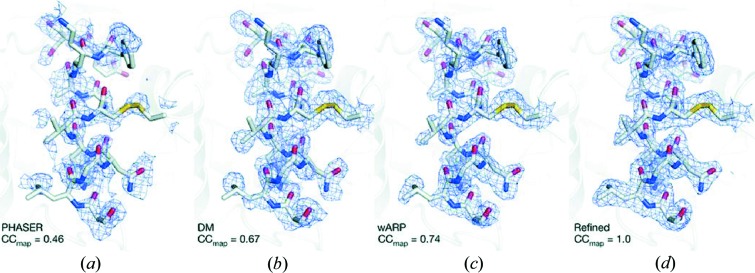
Electron density maps are shown. (*a*) Single-wavelength anomalous dispersion (SAD) phasing using the *PHASER* software (McCoy *et al.*, 2007 ▶). (*b*) Solvent flattening with the *DM* software package (Cowtan, 1994 ▶). (*c*) Automatic building using *wARP* (Langer *et al.*, 2008 ▶). (*d*) Final map after refinement. The correlation between the respective maps and the final, refined 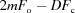 electron density *d* is indicated. All maps are contoured at 1.0σ. [Reprinted by permission from Macmillan Publishers Ltd: Barends *et al.* (2014 ▶), *Nature (London)*, **505**, 244–247, copyright (2014).]

In addition to the advantages involving crystal size, SFX also seeks to understand and ameliorate the problem of radiation damage, which has been the subject of much investigation and discussion. Early crystallography experiments established that the interaction of ionizing radiation with a crystal structure must inevitably lead to damage and disorder. Since then, so-called radiation damage has proved to be a significant challenge in obtaining high-resolution structural data. The tens-of-femtosecond pulse lengths of the FEL beam are thought to outrun radiation damage, but one critical bottleneck is that the models used for molecular replacement are based on synchrotron radiation measurements, which must still contend with damage. It is thus critical to be able to perform *de novo* phasing with SFX data in order to solve the structures of proteins for which no homologous structures are available. The feasibility of *de novo* phasing was demonstrated recently using lysozyme microcrystals soaked in a gadolinium compound (Barends *et al.*, 2014 ▶). Using 60000 diffraction patterns, a 2.1 Å resolution data set with a strong anomalous scattering signal was obtained, which were used to obtain phases using single-wavelength anomalous dispersion. After solvent flattening, the resulting experimental electron density map was good enough to allow automatic building of most of the structure (Fig. 3 ▶). These results pave the way for the structure determination of truly unknown proteins using SFX.

The extremely short pulse duration of LCLS induces damage mechanisms that are quite distinct from those experienced at synchrotron- and laboratory-based X-ray sources and, contrary to being a nuisance, can be the source of interesting new dynamics. The interaction of a crystal with the FEL pulse usually occurs on a time-scale of the order of tens of femtoseconds, which is too short for any significant motion of the nuclei to have occurred. However, motion of the electrons decoupled from the lattice is certainly possible on this time-scale as several experiments have demonstrated previously (Barty *et al.*, 2012 ▶). In general, it is expected that any dynamics of the electron density will be uncorrelated at large length scales and, therefore, will simply contribute a diffuse background signal. In an experiment originally designed to investigate the dynamical interactions of matter with intense FEL pulses, however, a user group observed that it is possible to induce, in crystalline C60 a long-range coherent reordering of the electron density of the crystal (Abbey *et al.*, 2012 ▶). This rearrangement of the electron density resulted in the formation of well defined Bragg peaks at locations distinct from the well known ground-state structure for C60. Effectively, a new electronic structure for the crystal has been formed on a femtosecond time-scale independent of any nuclear rearrangement; such a phenomenon has neither been reported nor predicted in any previously published work. Through modeling the quantum electrodynamics of the ionized C60 lattice, a plausible physical mechanism was established involving the long-range coupling of multiple dipoles to show how this new transient C60 structure is able to form on a femtosecond time-scale. Further investigation will be required to fully unravel the complex electron dynamics that occur during interaction of the crystal with the FEL pulse. The evidence so far strongly indicates that this is a new effect, only now revealed thanks to the unique capability of CXI to efficiently focus to sub-micrometer size, creating extremely high power densities on the samples.

**Figure 4:**
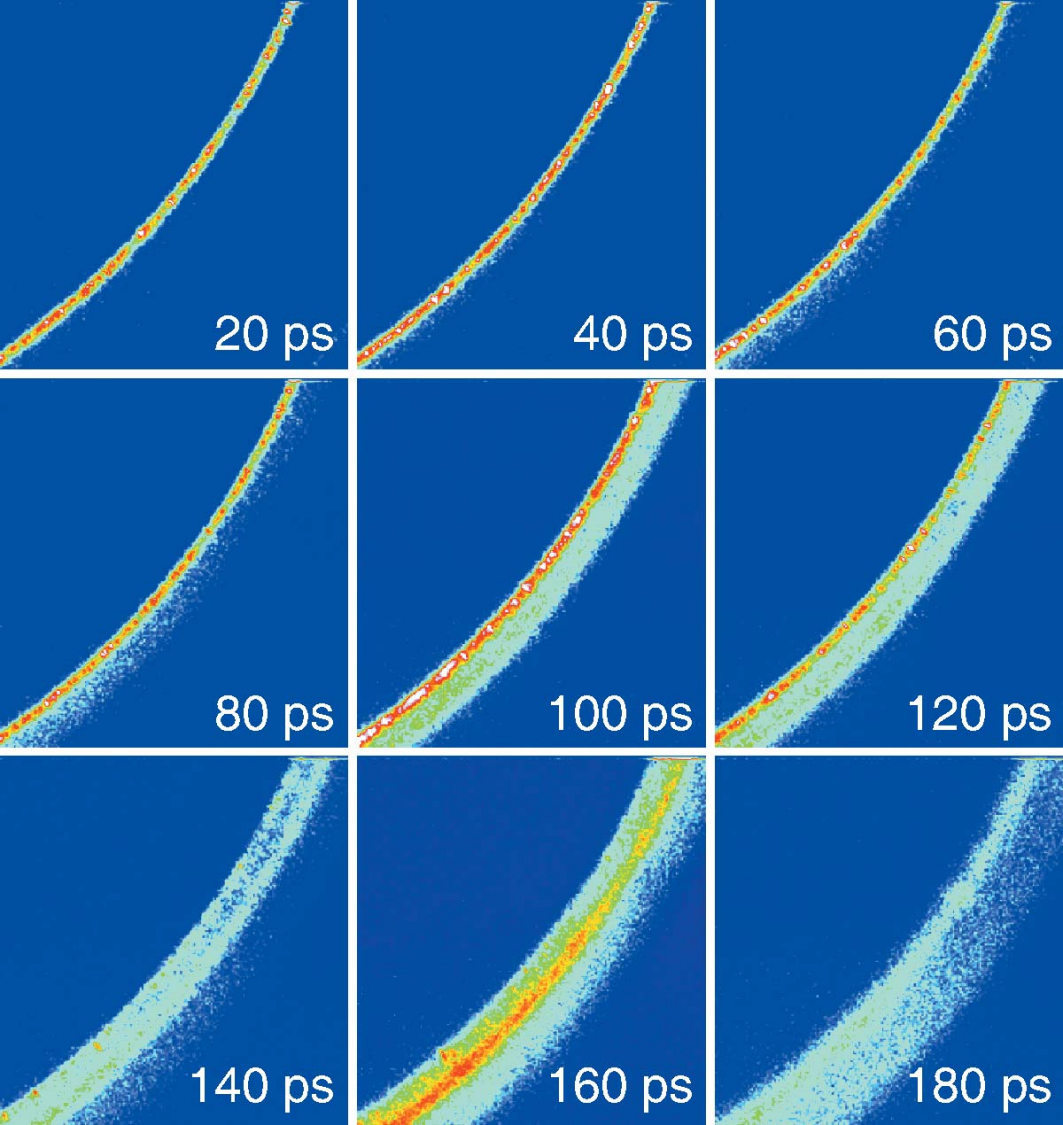
A section of the Cu (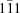) diffraction ring is magnified and its evolution is shown at 20 ps intervals. [From Milathianaki *et al.* (2013 ▶). *Science*, **342**, 220–223. Reprinted with permission from AAAS.]

The high intensity and short pulse length of CXI have also been used to study condensed matter systems. Milathianaki *et al.* (2013 ▶) studied the ultrafast response of a material to high pressures and strains at atomic length scales that would reflect the lattice dynamics in an experiment that took advantage of short pulse lengths. A pump–probe experiment with a 20 mJ, 170 ps Ti:sapphire laser was used to create shock waves in polycrystalline Cu films with peak elastic stresses of 73 GPa and strain rates of 10^9^ s^−1^. The copper foil was probed with a 30 µm × 30 µm X-ray probe with diffraction images taken at 10 ps intervals. Diffraction intensities, presented in Fig. 4 ▶ at 20 ps intervals, show the propagation of the shock wave as a redistribution of diffraction intensity from the unstrained lattice, to the elastically compressed lattice and then to the lattice under two-dimensional relaxation. The transition from a compressed to relaxed lattice state is indicative of a change from elastic, or reversible, deformation to permanent plastic deformation when the stress exceeds the elastic limit of the material. The results are in good agreement with molecular dynamics simulations which model the stress and strain values at these ultrafast time-scales (Milathianaki *et al.*, 2013 ▶). Such experiments demonstrate the flexibility of the CXI instrument and that of its laser capabilities.

## 4. Conclusion   

The Coherent X-ray Imaging (CXI) instrument at LCLS can perform a wide variety of hard X-ray experiments to take advantage of the unique capabilities of X-ray free-electron lasers. It is uniquely suitable for experiments which require high fluence in a vacuum environment and has yielded significant results across many areas of science. More details about the CXI instrument can be found at http://lcls.slac.stanford.edu/cxi

## 5. Facility access   

LCLS instruments are open to academia, industry, government agencies and research institutes worldwide for scientific investigations. There are two calls for proposals per year and an external peer-review committee evaluates proposals based on scientific merit and instrument suitability. Access is without charge for users who intend to publish their results. Prospective users are encouraged to contact instrument staff members to learn more about the science and capabilities of the facility, and opportunities for collaboration.

## Figures and Tables

**Table 1:** X-ray parameters and capabilities of the CXI instrument

Instrument name	CXI
Mirrors, maximum incidence angle	2 SiC on Si, 3.4mrad
Monochromaticity ( 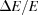 )†	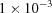 (SASE), 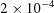 (seeded)
Energy range (keV)	511 (fundamental)
Unfocused beam size (m)	800 at 8.3keV
Focused beam size (m)	10, 1, 0.1
Focusing optics	Be lenses, one- and two-dimensional focusing
	Fixed Si 1m KB pair
	Fixed Si 100nm KB pair
Flux (photons per pulse)	1 10^12^ (fundamental‡)
Pulse length (fs)	5200, 40 nominal
Repetition rate (Hz)	120, 60, 30, 10, 5, 1, on demand
Optical laser pulse energy (mJ)	3 (800nm), 0.6 (400nm), 0.15 (266nm)
Optical laser pulse width (fs)	10150
Standard detectors	CSPAD (2), CSPAD-140k
Sample delivery	Aerodynamic lens injector for fine aerosols
	Gas dynamic virtual nozzle
	High-viscosity injectors
	In-vacuum fixed target stages
Spectrometer	Ion time-of-flight
	X-ray emission spectrometer

† Typical single-shot value.

‡ Excluding beamline and instrument transmission.
